# Supplementary material for: MetaRibo-Seq measures translation in microbiomes
Source: Nat Commun. 2020 Jun 29;11:3268. doi: 10.1038/s41467-020-17081-z (PMC7324362; doi:10.1038/s41467-020-17081-z)
Supplement: Supplementary file 10 — Supplementary Data 7 [file 41467_2020_17081_MOESM10_ESM.zip › File2/Confidence_High_Taxonomy/337564_out.krona.html]

Javascript must be enabled to view this page.

members
magnitude
magnitudeUnassigned
count
unassigned
taxon
rank

337564\_out

7

2
superkingdom
7

976
phylum
7

class
200643
7

171549
order
7

family
2005525
6

375288
genus
6


SRS019397\_contig\_number\_4097SRS023526\_contig\_number\_29510SRS043768\_contig\_number\_90SRS049995\_contig\_number\_24460SRS058145\_contig\_number\_3812SRS1041138\_contig\_number\_contig-100\_325.76552
6
823
species

family
815
1

816
genus
1

1

SRS077086\_contig\_number\_140
species
246787
